# Distribution of *Leishmania major *zymodemes in relation to populations of *Phlebotomus papatasi *sand flies

**DOI:** 10.1186/1756-3305-4-9

**Published:** 2011-01-25

**Authors:** Omar Hamarsheh

**Affiliations:** 1 Department of Biological Sciences, Al-Quds University, P.O. Box 51000, East Jerusalem, Palestine

## Abstract

*Phlebotomus papatasi *(Scopoli) (Diptera: Psychodidae) is the main vector of *Leishmania major *Yakimoff & Schokhor (Kinetoplastida: Trypanosomatidae), the causative agent of zoonotic cutaneous leishmaniasis in the Old World. Multilocus enzyme electrophoresis (MLEE) was extensively used to type different *L. major *stocks allover the world. Multilocus microsatellite typing (MLMT) has been recently used to investigate *P. papatasi *sand flies at population and subpopulation levels. In this article, the association between geographical distribution of *L. major *zymodemes and the distribution of populations and subpopulations of *L. major *vector; *P. papatasi *are discussed.

## Introduction

Zoonotic Cutaneous Leishmaniasis (ZCL) is caused by the parasitic protozoan *Leishmania major*, which is transmitted by the phlebotomine sand fly *Phlebotomus papatasi*. *P. papatasi *is also a vector of *Leishmania arabica *in Saudi Arabia [1,2], and of arboviruses in many countries [3,4]. Using various techniques like isoenzyme electrophoresis, polymerase chain reaction (PCR) and sequencing of different genes, *L. major *had been firmly identified in *P. papatasi *[5,6]. Many trypanosomatid infections have been recorded from wild sand flies [7]. However, many of these infections might have been other mammalian *Leishmania *species, or even lizard *Sauroleishmania*, as isolation and further characterization was not attempted. In countries north of Iran, much transmission of *L. major, L. turanica *and *L. gerbilli *is believed to occur in or near the burrows of the great gerbil *Rhombomys opimus *[8]. These burrows provide refuges for many sand fly species as well as for mammals and reptiles, making them a high risk habitat for the transmission of parasites to mammals and lizards [9]. In the former USSR, the distribution of *L. turanica *in rodents coincided with that of the sand flies *P. papatasi *[10]. The incrimination of a sand fly species as being the vector is complicated, Killick-Kendrick and Ward in 1981 established different grades of certainty; these include, anthropophily and geographic distribution in accord with the disease (Grade 1 and 2). Grade 3 requires that *Leishmania *be isolated from wild caught flies. Grade 4 establishes development of the parasite in the gut of the suspected vector. Grade 5 demonstrates experimental transmission of *Leishmania *by bite [11].

To date, there is no vaccine or effective cure for leishmaniasis [12]. It is postulated that successful establishment of the disease in an endemic area is the outcome of a close association between the *Leishmania *parasite and its natural sand fly vector. This close relationship between the parasite and its corresponding vector may have implications from the epidemiological point of view to the distribution of the disease as well as disease dynamics [13]. Close distribution results in considerable heterogeneity of both vector and parasite and this exemplifies a successful adaptation of a parasite resulting in distinct outcomes of infection and a complex epidemiological pattern. Several studies by leading researchers on sand fly- *Leishmania *interactions [14-17] proved a fairly selective relationship between *Leishmania *parasites and its corresponding sand fly vectors, which is typically the result of both ecological and molecular factors. This relationship involves the ability of *Leishmania *parasites to infect healthy sand flies by successful attachment of *Leishmanai *parasites to the sand fly midgut. In the case of *L. major*, attachment is provided by the parasite's surface lipophosphoglycan (LPG) to the Pp Galectines of the *P. papatasi *midgut proteins [15]. Other pathways existed; studies on mutants in LPG biosynthesis suggested the existence of LPG independent pathway of parasite attachment [18-20], thus, other unknown genes may potentially involve in the attachment relationship. Genetic variability among *Leishmania *and sand fly populations is crucial to unravel hidden genetic factors controlling the attachment of parasites to the midgut of the *P. papatasi *sand fly and therefore controlling the competence and infectivity of *P. papatasi *sand fly and *L. major *parasites respectively. To fulfill this long term goal, both *P. papatasi *and *L. major *were extensively studied and genetically typed in recent years using different molecular markers in a way to understand its systematics as well as population structures. These studies revealed the presence of isolated populations for both *L. major *and *P. papatasi *in different countries in the Old World [21-23].

Our knowledge about the wide genetic variability and phylogenetic relationships among the different lineages of *L. major *parasites and *P. papatasi *vectors has grown exponentially. This growth of knowledge has been supported by theoretical approaches based on population genetic analysis and the increasing development of molecular biology tools used to identify different genotypes. Genetically, *P. papatasi *is not a polymorphic sand fly. Phylogenetic studies using various molecular markers report a high genetic homogeneity [23,24].The level of genetic variability among *L. major *is very low and it is considered the least polymorphic *Leishmania *species [21,25].

Techniques of molecular biology including advancement in sequencing techniques are now becoming feasible and applicable in sand fly research [26-30]. On the other hand, parasite characterization were traditionally and routinely carried out by the French WHO reference laboratory at Montpellier (Laboratoire d'Ecologie Médicale et Pathologie Parasitaire, Faculté de Médecine, Montpellier, France) using multi locus enzyme electrophoresis, the zymodeme (defined as a collection of stocks that have the same isozyme profile [31] ) characterization based on starch gel multilocus enzyme electrophoresis (MLEE) typing. The zymodeme classification become popular over the years by providing a baseline insight into the molecular epidemiology of *L. major*. Since the distribution of zymodemes sometimes limited by geographical boundaries [32-34]. MLEE was very useful for the characterization of *L. major *parasites, but attempts to use MLEE to genetically differentiate *P. papatasi *populations which were carried out in the past provided no useful genetic information about the structure and distribution of different *P. papatasi *populations [35].

DNA markers such as genomic DNA sequence features and DNA microsatellites are useful for analyzing genetic variations among sand flies [23,36-39]. Mitochondrial DNA sequence polymorphism is very useful for studying differences between closely related species and among populations of the same species from different geographic locations, but it lacks the sensitivity to detect very subtle differences that frequently exist among very recently diverged populations [26,40]. The use of multilocus microsatellite analysis based on DNA sequences with short tandem repeat motifs such as (GT)_n _[41], demonstrates high variation in the number of repeats seen in individuals from different populations and thus are useful for studying population genetics [42]. Therefore, multilocus microsatellite typing (MLMT) has recently been applied to both *P. papatasi *sand flies and *L. major *parasites, providing additional information about the transmission dynamics of *L. major *[21,43] as well as the distribution and dispersal of *P. papatasi *[23].

In previous years, MLEE were used extensively to type isolates from various regions in Asia, Africa and Europe. The Laboratoire d'Ecologie Médicale et Pathologie Parasitaire, Faculté de Médecine, Montpellier, France became a reference for typing *Leishmania *isolates from all over the world. Zymodeme and DNA analysis proceeded in recent years in parallel lines; researchers used other genetic markers to reveal additional diversity within each zymodeme. Different molecular approaches have been used including random amplification of polymorphic DNA (RAPD), kDNA fragment length polymorphisms (schizodemes), karyotype variation, PCR amplicon size polymorphisms, PCR restriction fragment length polymorphisms (PCR-RFLPs) and comparative DNA sequencing of nuclear and mitochondrial targets [44-47]. In the literature there is much valuable information about the distribution of both *L. major *zymodemes [25] and *P. papatasi *sand fly populations and subpopulations [23,39]. Recently, a debate has been raised about the possible correlation between the distribution of populations and subpopulations of *P. papatasi *in relation to *L. major *zymodemes. Therefore, this review aimed to discuss the possible association between the distributions of *L. major *zymodemes in relation to the distribution of *P. papatasi *populations and subpopulations.

### *L. major *zymodemes versus *P. papatasi *populations

**Figure 1:**
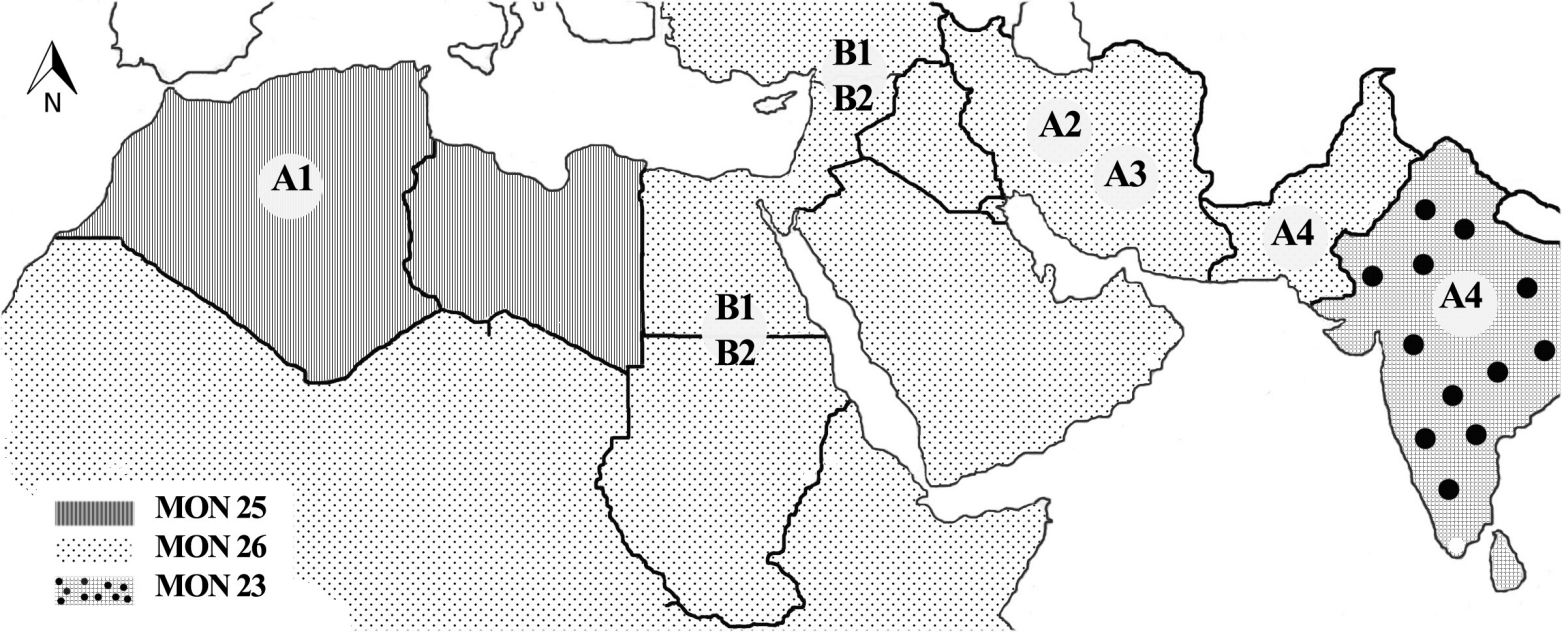
**Geographical distribution of *L. major *zymodemes in relation to *P. papatasi *populations and subpopulations**. Distribution of the main *L. major *zymodemes; MON 25, MON 26, MON 23 and MON 26 variants (MON 74, MON 103 and MON 117) in relation to the distribution of *P. papatasi *populations and subpopulations (A1-A4, B1, B2).

Globally, two main predominant and widespread zymodemes were reported for *L. major*, MON 25 and MON 26 [25]. More recently, a correlation between microsatellite markers and geographical origin was evaluated in *P. papatasi*, suggesting MLMT to be effective for population analysis in sand flies [23]. The geographical distribution of the main *L. major *zymodemes and populations of *P. papatasi *is shown in Figure 1.

In the east Mediterranean region (i.e., Palestine, Israel, Jordan, Turkey, and Syria), the commonest zymodeme is MON 26 which came from a semi-arid rural area where *Psammomys obesus *is widespread [25,48,49]. In agreement with MLEE, the population structures inferred by microsatellite typing of *P. papatasi *vector in these countries were mixed and restricted to only one population designated as 'B' which is further subdivided into 'B1'and 'B2'substructures without significant geographical distribution [23]. MON 26 is remarkably distributed over a large geographical range extending from Senegal in the west to Kazakhstan and Pakistan in the east. The east Mediterranean region represents small part of this range. Other less prevalent zymodemes include MON 66-68, MON 103 and MON 74 that were found to coexist in Palestinian Territories and Israel, Jordan and Egypt respectively. These less prevalent zymodemes considered as variants from MON 26 which only differ by a single enzyme each [25,32]. The *L. major *zymodeme in Sudan is MON 74, which considered as a variant from MON 26, and present within the range of MON 26, the same zymodeme was characterized from an *L. major *infected *P. duboscqi *sand fly collected from southern Ethiopia [50]. This is in agreement with the population structure of *P. papatasi *in Sudan in which 'B2' subpopulation is prevalent. MON 74 is also existed in southern Egypt, particularly in the areas close to the Sudanese borders; it seems that MON 74 diffused from Middle and Western Africa to Egypt, and probably other countries, where this zymodeme is less prevalent. This scenario is supported by historical information in which migration of people from Africa to the north was moved through Egypt.

The North Maghreb countries Tunisia, Algeria and Morocco share with a unique zymodeme MON 25, although other less prevalent zymodemes do exist; MON 24 in Tunisia and MON 269 in Morocco. The *P. papatasi *populations in Maghreb countries were exclusively belonging to 'A1' subpopulation [23]. To explain this discrepancy; MON 25 is a uniform zymodeme restricted to the Maghreb countries with high genetic stability. The presence of other less prevalent zymodemes in Maghreb (MON 269 which differs from MON 25 by single enzyme only (PGD)), may correspond to a recent mutation, or post-translational modification of the proteins that allow this slight change and the creation of new zymodemes [51]. The genetic homogeneity associated with the wide distribution of MON 25 suggests the emergence and rapid diffusion of this zymodeme over the whole Maghreb. MON 25 is diffused to the Sahel (Mali) which could possibly be explained by diffusion of the parasite due to human migration and/or to historical climatic changes (humid periods in Sahara) [25,52].

Using MLMT, *P. papatasi *populations in Iran were sub-structured in two subpopulations 'A2 and A3' with high population differentiation [23,53]. On the other hand mitochondrial and *Wolbachia *markers for *P. papatasi *showed little population differentiation between peridomestic sites and gerbil burrows in Isfahan province[37,54], even though the *L. major *parasites were belong to the same zymodeme; MON 26 that isolated from the eastern Middle East. It should be noted; based on isoenzyme characterization, *L. major *zymodeme MON 26 was identified in one infected *P. papatasi *[33] and in one infected *P. cuacasicus *sand flies [55]. This contradicted the population structure results, in which *P. papatasi *typed in this country, belong to two subpopulations (A2-A3) [23]. *L. major *zymodeme MON 26 has been isolated from both rodents and humans, and no other zymodemes were characterized in Iran [25,56-59]. Therefore, MLEE seems to be limited in detecting variations among *L. major *from Iran probably due to the fact that *L. major *has a limited isoenzyme polymorphism over a wide area in favor of poorly structured populations, or genetic variations were not affected the amino acid sequences in the studied enzymes in MLEE. Previous research documented the presence of genetic variations among different *L. major *strains from different provinces in the country. Using sequences from internal transcribed spacers, variations were manifested in five genotypic groups; LmA, LmB, LmC, LmD and LmE [22]. This could be the reason for the existence of new *L. major *endemic areas and increasing cases of cutaneous leishmaniasis which have been reported during recent years in Iran especially from border areas between Iran and Pakistan and between Iran and Afghanistan [60-63].

In India the prevalent zymodeme is MON 23 and *P. papatasi *population structure was depicted exclusively to 'A4'; there is a complete association between the distribution of zymodeme and population structure based on MLMT in this country [23,25]. In Pakistan, *P. papatasi *populations that originated from Baluchistan province was grouped in 'A4', this subpopulation includes, however, sand flies from India. No clear explanation from the differences between zymodeme distribution of *L. major *and population structure of *P. papatasi *in Pakistan is available, thus analysis of more sand flies and *Leishmania *stocks along the Iranian and Pakistani borders is needed. Baluchistan is a mountainous and arid area shared by Iran and Pakistan but divided by borders between the two countries. The prevalent zymodeme in this province is MON 26. This possibly explains that the presence of this zymodeme (MON 26) in this country may have originated from Iran, where MON 26 is widely spread.

## Conclusions and research needs

Among sand flies recorded from the Middle East, only *P. papatasi *was judged to be a proven vector of *L. major *[2]. The wide distribution range of *P. papatasi *is limited to a certain number of populations and subpopulations. This satisfies the wide distribution of *L. major *in the same range of *P. papatasi *which is also limited to a certain group of main zymodemes like MON 25, MON 26 and MON 23, and to other overlapping less prevalent variants. MON 25 is characterized in *P. papatasi *in Maghreb countries [64], the Middle-East, Saudi Arabia and Iran, MON 26 [33,65-68] and India, MON 23 [25].

This review provides baseline information about the association of MLMT and MLEE of the *P. papatasi *vector and *L. major *parasites. Further research is needed and should be directed toward analyzing more globally distributed parasite strains and sand fly samples using both MLMT and MLEE. Developing new polymorphic microsatellite markers for both *P. papatasi *sand flies and other *Leishmania *vectors is also essential. Studies and further investments in this field may disclose markers appropriate for population genetics and thus explain the vectorial capacity and parasite infectivity among its structures. Studies emphasizing the correlation between other sand fly species and with its correspondent vectors are very interesting research fields. The information obtained may significantly improve our understanding of the transmission and distribution of *Leishmania *parasites in relation to their vectors, and could possibly provide more information about sand fly- *leishmania *evolutionary relationships. This would have implications for leishmaniasis intervention and control. To address this long term and promising goal, it would be beneficial to create a public database that makes *Leishmania *and sand fly microsatellite allele data available worldwide. Finally, there is also a need for the standardization of the microsatellite genotyping techniques including the use of primers and the separation techniques.

## Competing interests

The author declares that he has no competing interests.
